# Catheter Ablation as a Treatment for Vasovagal Syncope: Long‐Term Outcome of Endocardial Autonomic Modification of the Left Atrium

**DOI:** 10.1161/JAHA.116.003471

**Published:** 2016-07-08

**Authors:** Wei Sun, Lihui Zheng, Yu Qiao, Rui Shi, Bingbo Hou, Lingmin Wu, Jinrui Guo, Shu Zhang, Yan Yao

**Affiliations:** ^1^ State Key Laboratory of Cardiovascular DiseaseCardiac Arrhythmia CenterFuwai HospitalNational Center for Cardiovascular DiseasesChinese Academy of Medical Sciences and Peking Union Medical CollegeBeijingChina; ^2^ Department of Cardiovascular MedicineThe First Affiliated HospitalXi'an Jiaotong University College of MedicineXi'anShaanxiChina

**Keywords:** autonomic modification, catheter ablation, ganglionated plexi, vasovagal syncope, Catheter Ablation and Implantable Cardioverter-Defibrillator

## Abstract

**Background:**

Autonomic modification through catheter ablation of ganglionated plexi (GPs) in the left atrium has been reported previously as a treatment for vasovagal syncope. This study aimed to observe the long‐term outcome in a larger cohort.

**Methods and Results:**

A total of 57 consecutive patients (aged 43.2±13.4 years; 35 women) with refractory vasovagal syncope were enrolled, and high‐frequency stimulation and anatomically guided GP ablation were performed in 10 and 47 cases, respectively. A total of 127 GP sites with positive vagal response were successfully elicited and ablated, including 52 left superior, 19 left lateral, 18 left inferior, 27 right anterior, and 11 right inferior GPs. During follow‐up of 36.4±22.2 months (range 12–102 months), 52 patients (91.2%) remained free from syncope. Prodromes recurred in 16 patients. No statistical differences were found between the high‐frequency stimulation and anatomically guided ablation groups in either freedom from syncope (100% versus 89.4%, *P*=0.348) or recurrent prodromes (50% versus 76.6%, *P*=0.167). The deceleration capacity, heart rate, and heart rate variability measurements demonstrated a reduced vagal tone lasting for at least 12 months after the procedure, with improved tolerance of repeated head‐up tilt testing. No complications were observed except for transient sinus tachycardia that occurred in 1 patient.

**Conclusions:**

Left atrial GP ablation showed excellent long‐term clinical outcomes and might be considered as a therapeutic option for patients with symptomatic vasovagal syncope.

## Introduction

Vasovagal syncope (VVS) is the most common etiology of a transient loss of consciousness in patients of all ages without apparent cardiac or neurological structural diseases.^1^, ^2^, ^3^, ^4^ Patients experiencing refractory syncopal episodes have poor quality of life and high risk of syncope‐related physical injuries.^5^, ^6^ Although the mechanism of VVS is not fully understood, an enhanced vagal tone via dysregulation of the Bezold–Jarisch reflex, together with a decreased sympathetic tone, contributes to cardioinhibitory and vasodepressor reactions in the pathogenesis of VVS.^7^

Treatment for VVS has been challenging. Conventional treatment (education, avoiding precipitating factors, and maintaining fluid and salt intake), orthostatic training, pharmacological treatments, and implantable rhythm devices have failed to show good clinical outcomes (25–65% recurrent syncope).^8^, ^9^, ^10^, ^11^, ^12^, ^13^

The search for a highly effective alternative therapy to modify the abnormally enhanced vagal tone over the long term is needed. Our early experience using endocardial catheter ablation to modify the ganglionated plexi (GPs) in the left atrium (LA) was reported in 10 human patients without any recurrent syncope during medium‐term follow‐up.^14^ The current study reports the long‐term results of this treatment in a larger patient population.

## Methods

### Patients

A total of 57 patients with frequent recurrent syncopal episodes were enrolled. All patients had (1) at least 3 episodes of syncope preceding the procedure or at least 1 syncopal episode within 6 months before recruitment; (2) positive response to head‐up tilt testing (HUT) at enrollment; and (3) failed conventional treatments including optimal fluid intake, physical counterpressure training, and pharmacological treatments. Failure was defined when spontaneous syncope recurred. Overall, 45 patients had been unresponsive to beta blockers (80 mg daily of propranolol or 50 mg daily of metoprolol). Two patients failed 0.2 mg daily of fludrocortisone, and 10 patients failed 10 mg daily of midodrine.

Exclusion criteria were as follows: (1) other causes of syncope including sinus node and atrioventricular block, hypertrophic cardiomyopathy, pulmonary hypertension, seizures, transient ischemic attacks, and subclavian steal syndrome; (2) severe comorbidities including myocardial infarctions within 6 months, New York Heart Association class III or IV heart failure, diabetes mellitus, or terminal disease; and (3) previous history of heart surgery, catheter ablation, or permanent pacemaker implantation. The local research ethics committee approved the study, and written informed consent was obtained from each participant at enrollment.

### Head‐up Tilt Testing

The detailed HUT protocol was described in our previous study.^14^ Patients were first tilted at 70° for 30 minutes (passive phase) and for an additional 20 minutes with 0.25 mg sublingually administered nitroglycerin (provocative phase) if no symptoms occurred during the passive phase. A positive response to the HUT was defined when syncope or the development of presyncope in the presence of bradycardia or hypotension occurred.^15^

### Preablation Preparation

All medications were discontinued for at least 5 half‐lives before the procedure. All procedures were performed under conscious sedation. Pulse oximetry and blood pressure were monitored during the procedure. Three right femoral venous accesses were obtained. A 6F decapolar steerable electrode catheter was placed in the coronary sinus, and a 6F quadripolar electrode catheter was positioned in the right ventricle apex. Intravenous heparin was prescribed after a single transseptal puncture to maintain an activated clotting time of 200 to 300 seconds. The 3‐dimensional geometry of the LA was created using an Ensite Array/NavX mapping system (St Jude Medical Inc). An 8‐mm‐tip deflectable catheter (Bard Electrophysiology or St Jude Medical) was used to deliver radiofrequency energy at the targeted sites. The power and temperature limits were 60 W and 60°C, respectively.

### High‐Frequency Stimulation–Guided Endocardial Catheter Ablation of the GPs in the LA

**Figure 1:**
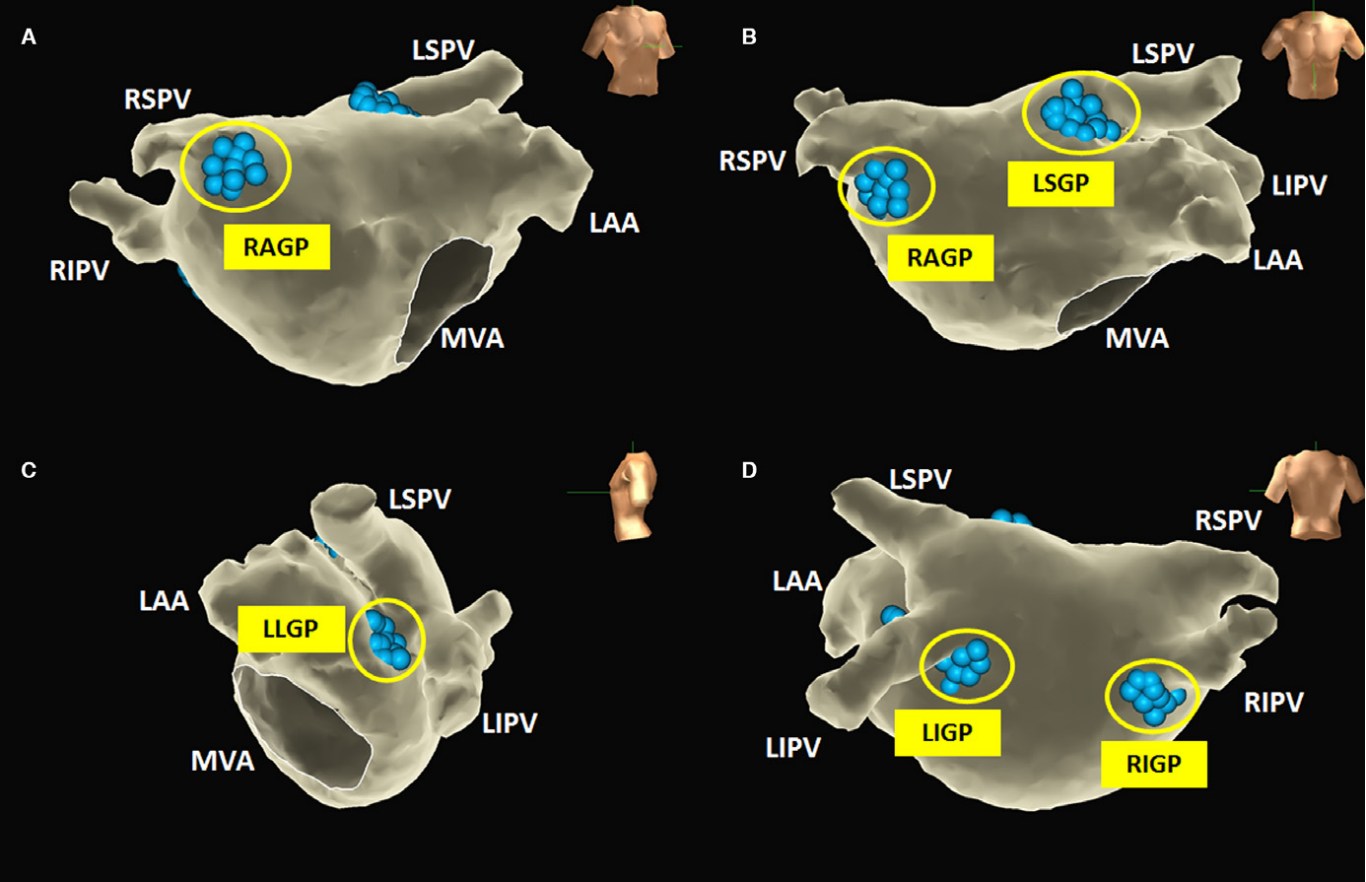
A 3‐dimensional computed tomography image of the left atrium showing the anatomical locations of the GPs. The blue balls represent the presumptive GP areas. A, The RAGP was well exposed in the right anterior oblique projection using electroanatomic guidance. B, The anteroposterior plus cranial projections helped identify the location of the LSGP. C, The LLGP between the LAA and LIPV was clearly exposed in the left lateral projection. D, The posteroanterior projection helped identify the locations of the LIGP and RIGP. GP indicates ganglionated plexus; LAA, left auricular appendage; LIGP, left inferior ganglionated plexus; LIPV, left inferior pulmonary vein; LLGP, left lateral ganglionated plexus; LSGP, left superior ganglionated plexus; LSPV, left superior pulmonary vein; MVA, mitral valve annulus; RAGP, right anterior ganglionated plexus; RIGP, right inferior ganglionated plexus; RIPV, right inferior pulmonary vein; RSPV, right superior pulmonary vein.

High‐frequency stimulation (HFS; 20 Hz, 10–20 V, pulse width 5 ms; MicroPace EPS320; Micropace EP) was applied to identify the GPs in the LA. Four GP sites were the particular focus of a previous report.^14^ Three‐dimensional electroanatomic guidance was highly recommended in this protocol because it provided detailed spatial information and different projection views to help identify the GP locations (Figure 1):

1. Left superior GP, located in the superolateral area around the root of the left superior pulmonary vein, was best exposed from the anteroposterior plus cranial projections.
2. Left inferior GP, located in the inferoposterior area around the root of the left inferior pulmonary vein, was best exposed from the posteroanterior projection.
3. Right anterior GP, located in the superoanterior area around the root of the right superior pulmonary vein, was best exposed from the right anterior oblique projection.
4. Right inferior GP, located in the inferoposterior area around the root of the right inferior pulmonary vein, was best exposed from the posteroanterior projection.

A positive vagal response (VR) was defined when HFS induced any of the following phenomena: transient ventricular asystole, atrioventricular block, or an increase in mean R‐R interval of 50%.^16^ In each presumptive GP site, HFS was delivered at 1 site for 2 to 5 seconds. If no VR was induced, another HFS was delivered at an adjacent site to form a cloudlike shape. If up to 5 consecutive HFS attempts failed to induce any VR, that GP was then defined as HFS negative. Radiofrequency ablation was performed at GP sites with positive VR. The end point of the ablation procedure was the elimination of all VRs at each identified target.

### Anatomically Guided Endocardial Catheter Ablation of GP in LA

**Figure 2:**
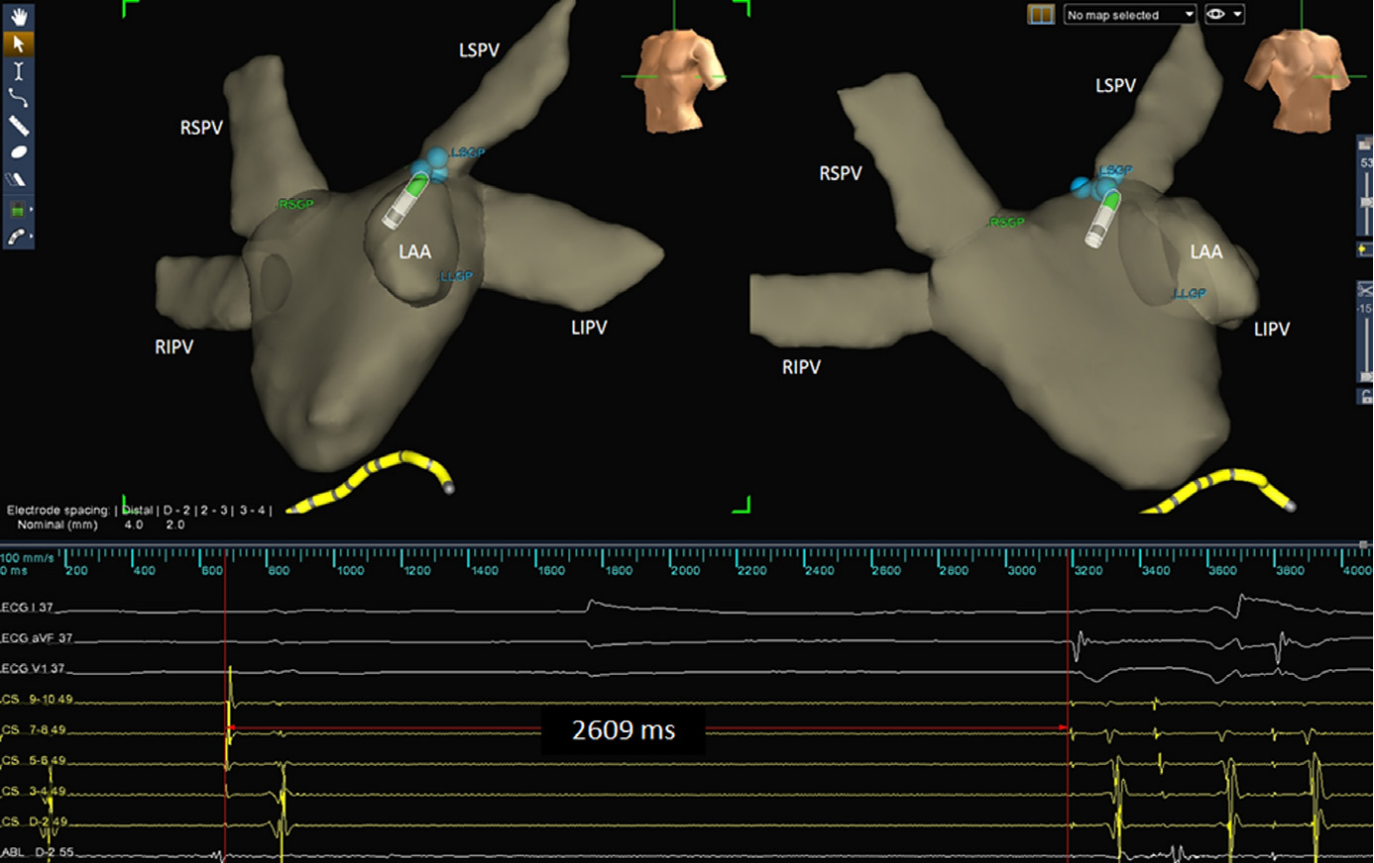
Illustration of the vagal response induced by radiofrequency energy delivery at the LSGP. The geometry of the left atrium was constructed using the Ensite Navx mapping system. The blue balls represent the ablated lesions with a positive vagal response at each GP site. The green dots represent the real‐time shadow of the catheter tip on the left atrial geometry. Radiofrequency ablation at the LSGP induced sinus arrest lasting 2609 ms. LAA indicates left auricular appendage; LIPV, left inferior pulmonary vein; LSGP, left superior ganglionated plexus; LSPV, left superior pulmonary vein; RIPV, right inferior pulmonary vein; RSPV, right superior pulmonary vein.

An anatomically guided GP ablation was carried out in 47 patients. In addition to the 4 GP sites specified, the left lateral GP—located between the left inferior pulmonary vein and the left auricular appendage—was integrated into the ablation protocol. The presumptive left lateral GP site was supposed to be near the ligament of Marshall, at which parasympathetic innervations were clustered.^17^ The GP sites were ablated sequentially from left superior to left lateral, left inferior, right anterior, and right inferior GPs (Figure 1). An illustration of the VR induced by radiofrequency energy is displayed in Figure 2. At each GP site, if tentative radiofrequency energy delivery induced any VR within 10 seconds, further energy was delivered for at least 30 seconds until inhibition of the VR. Otherwise the tentative ablation was terminated. Further ablation was then performed adjacent to the initial lesion to form a cloudlike lesion cluster. The end point of the anatomically guided procedure was defined as follows: Once at each GP site, 5 consecutive ablation attempts failed to induce any VR.

### Deceleration Capacity Analysis

As a quantitative evaluation of the cardiac vagal tone, the deceleration capacity (DC) was calculated using the phase‐rectified signal average technique introduced by Bauer et al.^18^ After acquiring the 12‐channel 24‐hour Holter ECG data, DC was generated automatically with the use of specific software (MIC‐12H Analysis Platform; Jinke Instruments). To assess the chronological effect of the GP ablation on the cardiac vagal tone, a DC analysis was carried out in 35 patients at the time of recruitment and repeated in each participant at 1 day and 1, 3, 6, and 12 months after ablation.

### Postablation Follow‐up

Previous medications including beta blockers, fludrocortisones, and midodrine were discontinued after the procedure. Postablation follow‐up consisted of a clinical visit (at 3, 6, and 12 months after ablation and contacted by telephone every 12 months), HUT (at 3 and 12 months after ablation), and Holter recording (scheduled at 3 and 12 months after ablation and repeated based on the patient's willingness for >12 months after ablation). Both recurrent syncope and any related physical injury were carefully documented. Prodromes including transient dizziness, diaphoresis, or fatigue without loss of consciousness were not considered recurrent episodes of syncope.

### Statistical Analysis

Continuous variables were reported as mean±SD for normally distributed data and as median (25–75% quartile) for nonnormally distributed data. Comparisons between the HFS‐ and anatomically guided groups were carried out using a Student *t* test (normally distributed data) or a Wilcoxon test (nonnormally distributed data). Categorical variables were reported as counts or as the number (percentage) of participants and compared by a Pearson chi‐square test or Fisher exact test. A 1‐sample paired *t* test was used to analyze the changes in heart rate and heart rate variability after the ablation procedure. Recurrent syncope or prodromes were compared using a Kaplan–Meier analysis. Repeated‐measures ANOVA was carried out to determine whether significant changes of DC occurred in patients with and without recurrent syncope over the course of 12 months after the procedure. Statistical significance was reached at a *P*<0.05. Statistical analyses were performed using SPSS software (version 19.0; IBM Corp).

## Results

### Patient Characteristics

In total, 57 patients (aged 43.2±13.4 years; 61.4% female) consented to participate in this study. The detailed demographic and clinical data of the enrolled participants are listed in Table 1. There were no statistical differences between the HFS‐guided (n=10) and anatomically guided (n=47) GP ablation groups. All patients apparently had normal hearts (LA 31.6±3.4 mm; left ventricular ejection fraction 64.3±4.0%). Before ablation, patients experienced a median of 9 episodes of syncope over a median of 3.0 years. In all, 61.4% of the participants experienced syncope‐associated trauma.

**Table 1:** Demographic and Clinical Information of the Enrolled Patients (n=57)

	Total (n=57)	HFS‐Guided Ablation (n=10)	Anatomically Guided Ablation (n=47)	*P* Value
Age, y	43.2±13.4	50.4±6.4	41.7±14.1	0.061
Sex, female (%)	35 (61.4)	7 (70)	28 (59.6)	0.725
Body mass index, kg/m^2^	22.3±2.6	22.6±1.7	22.2±2.8	0.723
Syncope history, y	3.0 (2.0–5.0)	2.0 (1.75–4.0)	3.0 (2.0–5.0)	0.331
Syncope burden				
Number of syncopal episodes in the preceding year	3 (2–5)	3.5 (3–6)	3 (2–5)	0.578
Total number of syncopal episodes	9 (4–15)	6.5 (3.75–10.0)	9 (5–16)	0.275
Patients who reported prodromes, n (%)	47 (82.4)	7 (70)	40 (85.1)	0.357
Patients who reported syncope‐associated trauma, n (%)	35 (61.4)	7 (70)	28 (59.6)	0.725
HUT				
Time to syncope during the HUT, minute	33.0 (16.5–38.5)	37.5 (31.25–40.0)	25.0 (16.0–37.5)	0.146
Systolic blood pressure decreased, mm Hg	36.1±27.1	48.3±24.2	33.5±27.1	0.116
Diastolic blood pressure decreased, mm Hg	27.4±17.9	36.2±15.5	25.5±17.9	0.087
Heart rate decreased, bpm	42.7±14.2	37.4±11.3	43.8±14.6	0.197
Supine hear rate, bpm	73.8±11.2	70.8±12.1	74.5±11.1	0.353
Supine systolic blood pressure, mm Hg	111.7±10.2	106.2±9.7	112.1±10.1	0.098
Supine diastolic blood pressure, mm Hg	69.2±8.5	65.7±8.9	70.0±8.3	0.087
Left atrium diameter, mm	31.6±3.4	32.3±3.5	31.5±3.4	0.491
Left ventricular ejection fraction, %	64.3±4.0	63.0±3.1	64.6±4.1	0.246

HFS indicates high‐frequency stimulation; HUT, head‐up tilt test.

### Endocardial GP Ablation in the LA

The detailed information of the procedures is listed in Table 2. Positive VRs were observed in 44.6% (127 of 285) of the GP sites. The left superior GP was the most common GP site at which a VR was seen (52 of 57, 91.2%). VRs were recorded at other GP sites: left lateral GP in 19 patients (33.3%), left inferior GP in 18 (31.6%), right anterior GP in 27 (47.4%), and right inferior GP in 11 (19.3%). The average number of GPs with a positive VR was 2.2±1.4 per person. The mean procedure time and fluoroscopy time were 44.8±6.3 and 7.0±3.3 minutes, respectively. The mean radiofrequency energy delivery time was 574.0±223.8 seconds. A detailed description of the radiofrequency applications and delivery times of the anatomically guided approach are shown in Table 2.

**Table 2:** Comparison Between the HFS‐ and Anatomically Guided Ablation Groups

	HFS‐Guided Ablation (n=10)	Anatomically Guided Ablation (n=47)	*P* Value
Procedure time, min	50.2±3.8	43.7±6.1	0.002
Fluoroscopy time, min	11.2±1.7	6.1±2.9	<0.001
RF applications, n	9.1±1.4	34.6±5.4	<0.001
LSGP	6.3±0.9	11.0±3.2	<0.001
LLGP	—	6.2±1.7	—
LIGP	1.5±2.0	5.9±1.6	<0.001
RAGP	1.3±1.4	6.1±1.6	<0.001
RIGP	0	5.4±0.9	<0.001
RF delivery time, s	345.0±121.9	622.8±210.3	<0.001
LSGP	242.0±73.3	275.1±117.7	0.398
LLGP	—	97.2±68.5	—
LIGP	54.0±75.9	88.1±61.7	0.134
RAGP	49.0±56.1	93.4±59.1	0.034
RIGP	0	68.9±40.6	<0.001
Positive vagal response observed at each GP			
LSGP	10/10	42/47	0.574
LLGP	—	19/47	—
LIGP	3/10	15/47	1.000
RAGP	5/10	22/47	1.000
RIGP	0/10	11/47	0.183

GP indicates ganglionated plexus; HFS, high‐frequency stimulation; LIGP, left inferior ganglionated plexus; LLGP, left lateral ganglionated plexus; LSGP, left superior ganglionated plexus; RAGP, right anterior ganglionated plexus; RF, radiofrequency; RIGP, right inferior ganglionated plexus.

Compared with the HFS‐guided group (n=10), the patients in the anatomically guided group (n=47) had a shorter procedure time (*P*=0.002), a shorter fluoroscopy time (*P*<0.001), and a longer radiofrequency energy delivery time (*P*<0.001). No statistical difference was observed between the 2 groups regarding the VR induced at each GP site.

### Clinical Outcomes

**Figure 3:**
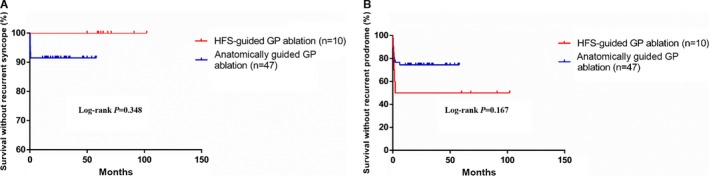
Kaplan–Meier curves of recurrent syncope and recurrent prodromes in HFS‐ and anatomically guided ablation groups. A, Syncope recurrence in HFS‐ and anatomically guided ablation groups (0% vs 10.6%, *P*=0.348). B, Prodrome recurrence in HFS‐ and anatomically guided ablation groups (50% vs 23.4%, *P*=0.167). GP indicates ganglionated plexus; HFS, high‐frequency stimulation.

For the total cohort (n=57), during an average follow‐up period of 36.4±22.2 months (range 12–102 months), syncope and prodromes recurred in 5 and 16 participants, respectively. Recurrent syncope events did not differ between the HFS‐ and anatomically guided ablation groups (0% versus 10.6%, *P*=0.348). Furthermore, survival analysis revealed no statistical difference between the 2 groups regarding recurrent prodromes (*P*=0.167) (Figure 3).

The 5 patients with recurrent syncope were the same participants in whom a positive VR could not be induced at any GP site. The mean time to first recurrent syncopal episode after ablation was 10.0±5.4 days (range 2–17 days), and the 5 patients had 9.0±6.7 episodes (range 2–17 episodes) of syncope within 1 year after the procedure. Syncope‐related physical injury occurred in 1 male patient and caused facial trauma necessitating surgical assistance. Although prodromes recurred in 16 patients within an average time of 34.2±42.3 days (range 3–182 days) after ablation, obvious symptomatic alleviation was reported.

Transient sinus tachycardia occurred in 1 female patient, but she recovered within 3 days before discharge. There were no other procedure‐related complications, including vascular access events, tamponade, pericarditis, or symptoms related to a delay in gastric emptying.

### DC, Heart Rate, Heart Rate Variability, and HUT Changes After Denervation

**Figure 4:**
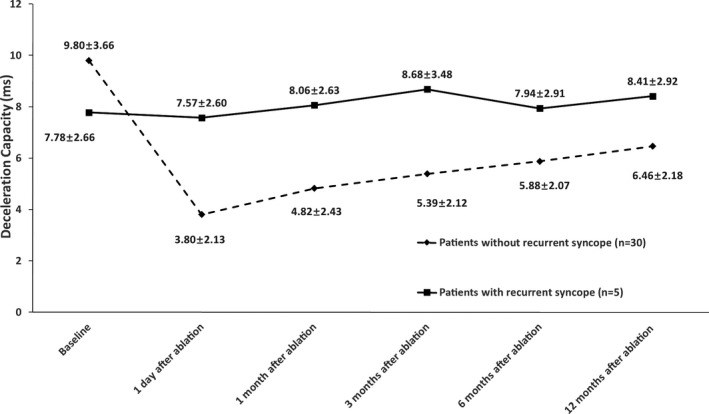
Chronological changes in the deceleration capacity in patients with (n=5) and without (n=30) recurrent syncope.

The chronological changes in DC are displayed in Figure 4. According to repeated‐measures ANOVA, there were significant differences in DC between patients with and without recurrent syncope (F=7.927, *P*=0.008). In addition, there was a significant time‐course effect (F=11.864, *P*<0.001) and strong interaction between time‐course and group (F=2.859, *P*=0.026) in DC measurements (Table 3).

**Table 3:** Effect of Group (With or Without Recurrent Syncope) and Time Course (5 Follow‐up Visits) on DC (Repeated‐Measures ANOVA)

Source	Numerator, *df*	F	*P* Value
Intercept	1	166.787	<0.001
Group	1	7.727	0.008
Time course	4	11.864	<0.001
Time course×Group	4	2.859	0.026

DC indicates deceleration capacity.

The HUT was repeated in all patients 3 months after the procedure and was positive in 20 patients. The average time to onset of symptoms during the HUT was delayed by 10.8±7.6 minutes (*P*=0.008), and in 6 patients, the syncope was postponed from the passive phase to the nitroglycerin provocative phase. Twelve patients refused to undertake a 12‐month postablation HUT. In the remaining 45 patients, 13 patients exhibited positive reactions to the HUT, among which 3 patients were negative at the 3‐month postablation HUT. In the remaining 10 patients, 80% of the positive reactions were provoked during the nitroglycerin phase.

Heart rate variability and heart rate demonstrated significant changes at 3 months that persisted at 12 months after the procedure (Table 4). Compared with the baseline measurement, the time‐ and frequency‐domain heart rate variability was significantly lower (except at low frequency), whereas the minimum, mean, and maximum heart rates were significantly higher (*P*<0.01). After an average period of 28.7±9.8 months after ablation, only the minimum heart rate remained higher than before the ablation (*P*=0.022).

**Table 4:** Long‐Term Changes in HR and HR Variation After Ablation

	Before Ablation (n=57) Mean±SD	3 Months After Ablation (n=57)		12 Months After Ablation (n=57)		28.7±9.8 Months After Ablation (n=40)	
		Mean±SD	*P* Value^a^	Mean±SD	*P* Value^a^	Mean±SD	*P* Value^a^
SDNN, ms	129.2±14.1	112.0±11.9	<0.001	105.3±17.2	<0.001	126.7±13.7	0.077
rMSSD, ms	29.4±9.2	19.8±8.2	<0.001	24.3±8.3	<0.001	28.6±7.2	0.175
In LF, ms^2^	5.54±0.49	5.06±0.47	<0.001	5.48±0.35	0.354	5.52±0.73	0.736
In HF, ms^2^	5.12±0.25	4.73±0.29	<0.001	4.71±0.35	<0.001	4.99±0.97	0.392
Minimum HR, bpm	48.6±6.5	61.1±6.5	<0.001	54.2±6.6	<0.001	50.9±5.6	0.022
Maximum HR, bpm	120.0±11.3	128.5±11.8	<0.001	123.9±8.0	0.016	118.1±9.2	0.875
Mean HR, bpm	65.6±5.8	74.0±6.2	<0.001	70.9±7.2	<0.001	65.8±5.3	0.751

HF indicates high frequency; HR, heart rate; LF, low frequency; rMSSD, root mean square of successive differences; SDNN, standard deviation of all N‐N intervals.

a Compared with before ablation.

## Discussion

### Major Findings

The results of this long‐term clinical follow‐up revealed the following findings: (1) HFS‐ and anatomically guided catheter ablation of GPs in the LA could effectively prevent recurrent spontaneous syncopal episodes in patients suffering from refractory VVS (91.2% free from syncope at 102 months after ablation); (2) compared with the HFS‐guided ablation strategy, anatomically guided ablation achieved an identical curative effect while significantly decreasing the procedure and fluoroscopy time; (3) the effect of the LA GP ablation on cardiac autonomic modulation, especially on vagal tone, persisted at least 12 months after ablation, as shown by repeated DC analysis, heart rate variability measurements, and HUTs.

### Previous Studies Adopting Catheter Ablation as a Treatment for Neural Mediated Syncope

In 2006, Pachon et al^19^ reported that spectral mapping–guided vagal denervation achieved symptomatic relief in 6 patients with neural mediate syncope during an average follow‐up of 9 months. They updated their research in 43 patients with important cardioinhibition during a HUT in 2011.^20^ Three patients experienced recurrent syncope in 45.1±22 months, and all postablation repeated HUTs were negative except for 4 partial cardioinhibitory responses. Scanavacca et al^21^ applied selective vagal denervation of the atrioventricular nodes and the sinus node to treat a female patient aged 15 years, and the syncope recurred 9 months later. Liang et al^22^ performed an HFS‐guided atrial vagal ablation (near coronary sinus ostium and super vena cava and at the posterior wall of the pulmonary vein antrum) in a woman aged 57 years and achieved 12‐month survival without any syncope. Rebecchi et al^23^ presented an anatomically guided ablation of the supero‐, mid‐, and inferoposterior right atrium in 2 patients. Syncope recurred in 1 patient 5 months later and was absent in 1 patient during an 8‐month follow‐up period.

### HFS‐ and Anatomically Guided LA GP Ablation in Our Study

Unlike the previous studies, the HFS‐ and anatomically guided ablation protocols were carried out exclusively in the LA instead of in the right atrium or interatrial septum. The HFS‐guided LA GP ablation strategy in our preliminary experience was developed based on histological and anatomic findings in both animals and human patients. First, the parasympathetic nerves are distributed more densely than sympathetic nerves in the atrium, with a ratio between 1.3 and 1.6, and are located mainly in the subendocardial area of the myocardium.^24^, ^25^ Second, anatomic studies of the intrinsic cardiac nerve system suggested that the GPs in the LA are located mainly around the root of the pulmonary veins.^26^, ^27^, ^28^, ^29^ These features made it possible to modify cardiac autonomous innervation through endocardial catheter ablation.

Because we observed that the distribution of the GPs in the LA was comparatively fixed and that a VR could also be induced by radiofrequency energy delivery during ablation, the anatomically guided ablation strategy was advanced. To achieve a balance between precise GP localization and limited unexpected ablation injury, a method of delivering multiple tentative radiofrequencies was introduced. Interestingly, the induction rate of VRs induced by radiofrequency varied greatly in the different studies. Po et al^16^ pointed out that the induction rate of VRs by radiofrequency was rather low, whereas Pappone et al^30^ reported an induction rate of VRs by radiofrequency of 95% at the junction between the left superior pulmonary vein and the LA, 70% between the left inferior pulmonary vein and the LA, 50% between the right inferior pulmonary vein and the LA, and 25% between the right superior pulmonary vein and the LA; those findings were compatible with ours. As far as we are concerned, the differences in the VR induction rate could be explained by the different patient populations (atrial fibrillation and VVS) and the heterogeneity among the patients.

Compared with the HFS‐guided ablation strategy, anatomically guided ablation exhibited shorter procedure and fluoroscopy times (*P*<0.01) and achieved an identical success rate in preventing recurrent syncope (*P*=0.348). A possible explanation for this reduction in the procedure and fluoroscopy times is that the HFS‐guided approach required extra time to restimulate the ablated targets to verify whether the VRs had been eliminated. Moreover, less radiation exposure was needed to establish the electroanatomic LA geometry as the operator gained experience.

### Rationale for LA GP Ablation in Treating VVS

Previous reports found that the GPs were important components of the cardiac intrinsic autonomous system.^28^, ^31^, ^32^ They receive inputs from both mechanoreceptors and chemosensory receptors and participate in the interaction between the efferent and afferent neurons of the cardiac autonomous system.^33^ We speculated that denervation of the GPs was capable of breaking both the efferent and afferent pathways of the abnormal Bezold–Jarisch reflex. On the one hand, the ablated GPs prevented the mechanoreceptor‐ or chemoreceptor‐mediated impulses, which were induced by emotional stimuli or a decreased venous return, from being transmitted into the medullary vasomotor center. This influence on the afferent pathway avoided the initiation of the abnormal Bezold–Jarisch reflex. On the other hand, the efferent vagal inputs to the heart (causing bradycardia and atrioventricular conduction block) were inactivated by the denervation of the GPs. This interference on the efferent pathway prevented the bradycardia and hypotension (low cardiac output was a cause of the decreased blood pressure), which resulted in preventing recurrent syncope.

### Long‐Term Clinical Outcome and Vagal Denervation

In the current study, the survival rate without recurrent syncope was 91.2% during an average follow‐up period of 36.4±22.2 months. The long‐term clinical outcome was promising compared with previous reports with conventional treatments, tilt training, pharmacological treatments, or pacemaker implantation (35–75% free from recurrent syncope).^8^, ^9^, ^10^, ^11^, ^12^, ^13^ Although the previous studies in animals suggested that ablation of the GPs might increase the vulnerability to atrial or ventricular arrhythmias,^34^, ^35^, ^36^ there was only 1 case of quickly recovered sinus tachycardia in the current study. Nevertheless, the proarrhythmic effect of the GP ablation still needs to be addressed regarding the candidates and should be followed carefully.

The medium‐ and long‐term effects of vagal denervation by catheter ablation have not been fully elucidated. In our study, significant changes in heart rate and heart rate variability and better tolerance of the HUT persisted for at least 12 months after the procedure. Furthermore, the DC changes differed between patients with and without recurrent syncope, according to repeated‐measures ANOVA. Heart rate and heart rate variability, however, returned to baseline after a long period of time, and there was a discrepancy between the autonomic parameters and the clinical outcome that was also observed in other studies.^20^, ^23^ It is surmised that although reinnervation occurred and the parasympathetic and sympathetic activity rebalanced during long‐term follow‐up, endocardial ablation of the GPs modified the excessive parasympathetic reactions as a result of dysregulation of the Bezold–Jarisch reflex, which in turn prevented recurrent syncope.

### Study Limitations

Although the current study has a greatly augmented patient population size compared with the preliminary report, this was still a prospective, single‐center, nonrandomized study. It is acknowledged that the small sample size in the current study limited the power to find significant results. To minimize the placebo effect brought on by the invasive approach, comprehensive communication was established with the patients to ensure that they were fully aware of the potential complications and the probability that they could not benefit from this research. Randomized and controlled trials are necessary to confirm this therapeutic approach.

## Conclusion

HFS‐ and anatomically guided LA GP ablation succeeded in preventing spontaneous recurrent syncope during long‐term follow‐up (91.2% without syncope at 102 months after the procedure). Catheter ablation appears to be an effective and safe treatment option for patients with refractory VVS. Further randomized controlled trials are required to promote this alternative treatment.

## Sources of Funding

This work was supported by the Beijing Municipal Science & Technology Commission (Z121107001012014).

## Disclosures

None.
